# Characterization of a novel mouse model with genetic deletion of CD177

**DOI:** 10.1007/s13238-014-0109-1

**Published:** 2014-10-31

**Authors:** Qing Xie, Julia Klesney-Tait, Kathy Keck, Corey Parlet, Nicholas Borcherding, Ryan Kolb, Wei Li, Lorraine Tygrett, Thomas Waldschmidt, Alicia Olivier, Songhai Chen, Guang-Hui Liu, Xiangrui Li, Weizhou Zhang

**Affiliations:** 1 College of Veterinary Medicine, Nanjing Agricultural University, Nanjing, 210095 China; 2 Department of Pathology, Holden Comprehensive Cancer Center, Carver College of Medicine/University of Iowa, Iowa, IA 52242 USA; 3 Department of Internal Medicine, Carver College of Medicine/University of Iowa, Iowa, IA 52242 USA; 4 Department of Pharmacology, Carver College of Medicine/University of Iowa, Iowa, IA 52242 USA; 5 National Laboratory of Biomacromolecules, Institute of Biophysics, Chinese Academy of Sciences, Beijing, 100101 China; 6 Beijing Institute for Brain Disorders, Beijing, 100069 China

**Keywords:** CD177, neutrophil, mouse model, genetic deletion

## Abstract

Neutrophils play an essential role in the innate immune response to infection. Neutrophils migrate from the vasculature into the tissue in response to infection. Recently, a neutrophil cell surface receptor, CD177, was shown to help mediate neutrophil migration across the endothelium through interactions with PECAM1. We examined a publicly available gene array dataset of CD177 expression from human neutrophils following pulmonary endotoxin instillation. Among all 22,214 genes examined, CD177 mRNA was the most upregulated following endotoxin exposure. The high level of CD177 expression is also maintained in airspace neutrophils, suggesting a potential involvement of CD177 in neutrophil infiltration under infectious diseases. To determine the role of CD177 in neutrophils *in vivo*, we constructed a CD177-genetic knockout mouse model. The mice with homozygous deletion of CD177 have no discernible phenotype and no significant change in immune cells, other than decreased neutrophil counts in peripheral blood. We examined the role of CD177 in neutrophil accumulation using a skin infection model with *Staphylococcus aureus*. CD177 deletion reduced neutrophil counts in inflammatory skin caused by *S. aureu*s. Mechanistically we found that CD177 deletion in mouse neutrophils has no significant impact in CXCL1/KC- or fMLP-induced migration, but led to significant cell death. Herein we established a novel genetic mouse model to study the role of CD177 and found that CD177 plays an important role in neutrophils.

**Electronic supplementary material:**

The online version of this article (doi:10.1007/s13238-014-0109-1) contains supplementary material, which is available to authorized users.

## INTRODUCTION

*CD177* is a polymorphic gene that has been linked to several important clinical diseases including polycythemia vera, Wegner’s granulomatosis, and immune mediated neonatal neutropenia (Lalezari et al., 1971; Bettinotti et al., 2002; Caruccio et al., 2006). The NB1/CD177 glycoprotein was initially identified in several cases of neonatal neutropenia (Lalezari et al., 1971), where the maternal antibodies react specifically with neonatal neutrophil-specific epitope HNA-2a derived from NB1 glycoprotein. In several cases of transfusion-related acute lung injury, CD177-specific antibodies from donor blood were identified as the primary cause (Bux et al., 1996). CD177 has been established as a diagnostic marker for various myeloproliferative diseases including polycythemia vera, thrombocythemia, and idiopathic myelofibrosis (Stroncek et al., 2004; Sirhan et al., 2005; Martini et al., 2006; Michiels et al., 2007). This 58–64 kDa glycophosphatidylinositol (GPI)-linked N-glycosylated extracellular surface protein (Goldschmeding et al., 1992; Dillon et al., 2008) is expressed on a subpopulation of neutrophils (Matsuo et al., 2000). In general, 20%–80% of circulating neutrophils are CD177 positive, with 3%–5% of population having no CD177^+^ neutrophils (Matsuo et al., 2000).

CD177 belongs to the uPAR/CD59/Ly6 snake toxin superfamily and is conserved in many species (Kissel et al., 2001; Stroncek, 2007). While percentages of CD177^+^ circulating neutrophils are constant in the same individuals (Goldschmeding et al., 1992; Stroncek et al., 1998), CD177 mRNA and protein expression are increased in response to inflammatory stimulation. Granulocyte colony stimulating factor (G-CSF) strongly induces CD177 expression (Stroncek et al., 1998). Expression of CD177 is also altered during pregnancy (Caruccio et al., 2003) and during severe bacterial infections (Gohring et al., 2004). The function of CD177 in neutrophil biology is largely unknown. Recent literature indicates that CD177 mediates the migration of neutrophils across endothelial cells via interactions with proteinase 3 (Kuckleburg et al., 2012; Kuckleburg and Newman, 2013) and PECAM-1 (CD31) (Sachs et al., 2007; Bayat et al., 2010), as well as the degranulation and superoxide generation in a Mac-1-dependent manner (Jerke et al., 2011). These *in vitro* studies suggest a role for CD177 in neutrophil transmigration. However, a recent study indicated that CD177^-^ and CD177^+^ neutrophils accumulate similarly in the peritoneal cavity of human peritonitis patients (Wang et al., 2013), suggesting that CD177 expression in neutrophils provides no transmigration advantage into this site.

Using a murine CD177 knockout model, the present study demonstrates that CD177 plays a crucial role in neutrophil viability, but has no impact on chemotaxis.

## RESULTS

### CD177 mRNA expression is increased in human neutrophils following pulmonary endotoxin instillation and in neutrophils isolated from septic patients

**Figure 1:**
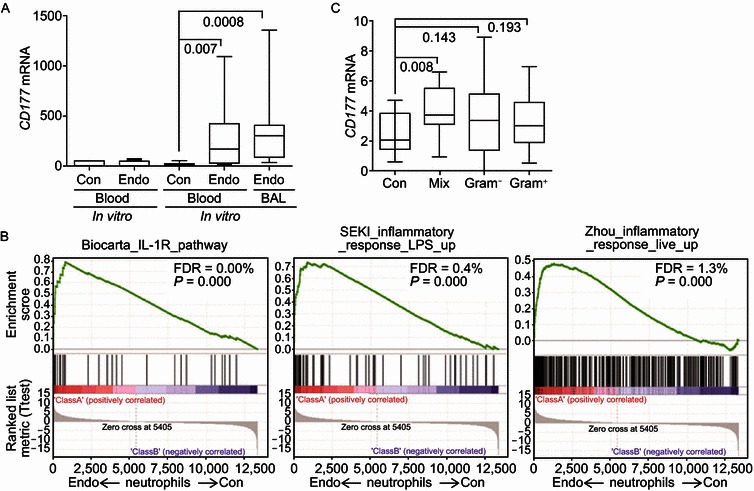
**Pulmonary endotoxin instillation induces CD177 expression in circulating and airway neutrophils**. (A) GEO dataset GSE2322 was downloaded and analyzed for endotoxin-induced CD177 mRNA expression from purified neutrophils before and after *in vitro* endotoxin treatment, circulating neutrophils before *in vivo* endotoxin treatment, or circulating neutrophils and airway neutrophils after *in vivo* endotoxin treatment. *n* = 14 for circulating neutrophils prior to endotoxin treatment; *n* = 17 for circulating neutrophils post endotoxin treatment; *n* = 17 for airway neutrophils post endotoxin treatment. (B) Gene Set Enrichment Analysis (GSEA) was performed to compare biological pathways that are different between circulating neutrophils before and after endotoxin instillation, or between circulating neutrophils before endotoxin instillation and airway neutrophils after endotoxin treatment. Both distinct pathways and common pathways are summarized in supplemental Tables 1–3. Common pathways are shown including IL-1R signaling, LPS-induced inflammatory pathway, and live *Porphyromonas gingivalis*-induced inflammatory pathway. (C) GEO dataset GSE5772 was downloaded including 17 non-infected normal controls, 25 septic patients infected with gram-negative bacteria, 18 septic patients infected with gram-positive bacteria, and 12 septic patients with both gram-negative and gram-positive bacterial infections; CD177 expression is in log_2_. (A and C) *P* values are shown; (B) Both *P* values and false discovery rate (FDR) are shown

To understand how neutrophils respond to bacterial stimulus at the transcriptional level, we analyzed a published microarray dataset (GSE2322) (Coldren et al., 2006) using circulating neutrophils before and after bacterial endotoxin (LPS) instillation, and neutrophils from bronchoalveolar lavage (BAL) after endotoxin instillation from human volunteers. Endotoxin treatment alone did not induce CD177 expression in purified blood neutrophils *in vitro* (Fig. 1A). However, CD177 is the most significantly upregulated gene (13 fold induction) in circulating neutrophils among all the genes induced by endotoxin *in vivo* (Table 1 and Fig. 1A). Another 13 genes were co-upregulated with CD177 with a more than two fold increase, and *P* values less than 0.05 (Table 1). The expression of CD177 mRNA was also high after pulmonary endotoxin exposure (Table 1 and Fig. 1A). The significant upregulation of CD177 in both circulating and airway neutrophils implies an important role for CD177 in neutrophil function in response to bacterial infection in the lung. As expected, pulmonary endotoxin exposure leads to significantly elevated pro-inflammatory pathways identified by gene sets enrichment analysis (GSEA) (Subramanian et al., 2005), including the IL-1R pathway, TH1/TH2 pathway, LPS-induced inflammatory pathway and other pathways related to inflammation (Fig. 1B, Tables S1–3).

**Table 1:** Genes were induced by pulmonary endotoxin treatment

Probe_ID	Gene symbol	Blood Neu after/before endotoxin		BAL Neu/Blood Neu	
		Fold change	*P* value	Fold change	*P* value
219669_at	CD177	12.98	4.11 × 10^−3^	20.34	4.34 × 10^−3^
203021_at	SLPI	3.48	1.28 × 10^−3^	9.11	1.49 × 10^−5^
207500_at	CASP5	3.25	5.98 × 10^−3^	15.66	1.29 × 10^−4^
204860_s_at	NAIP	3.05	8.37 × 10^−4^	4.03	1.33 × 10^−2^
209369_at	ANXA3	2.97	2.71 × 10^−3^	4.48	6.84 × 10^−6^
208771_s_at	LTA4H	2.58	6.06 × 10^−4^	4.20	1.79 × 10^−3^
200985_s_at	CD59	2.55	7.02 × 10^−3^	3.94	3.44 × 10^−5^
210166_at	TLR5	2.55	2.80 × 10^−4^	2.99	4.67 × 10^−3^
200984_s_at	CD59	2.53	1.40 × 10^−3^	3.60	2.48 × 10^−5^
203233_at	IL4R	2.51	1.28 × 10^−4^	2.34	6.66 × 10^−4^
201554_x_at	GYG1	2.45	1.49 × 10^−3^	3.60	2.60 × 10^−7^
217823_s_at	UBE2J1	2.27	7.10 × 10^−4^	2.30	1.19 × 10^−4^
209835_x_at	CD44	2.22	2.53 × 10^−3^	21.12	5.31 × 10^−10^
206697_s_at	HPR	2.14	8.11 × 10^−3^	2.61	2.89 × 10^−2^
212135_s_at	ATP2B4	2.09	4.05 × 10^−5^	2.29	1.78 × 10^−3^
204232_at	FCER1G	2.05	6.75 × 10^−4^	4.07	1.47 × 10^−7^
212136_at	ATP2B4	2.01	3.80 × 10^−3^	2.32	5.98 × 10^−3^

GEO dataset GSE2322 was downloaded including transcripts from circulating neutrophils and bronchoalveolar lavage neutrophils obtained from human volunteers before and after endotoxin instillation. Genes that were significantly induced by endotoxin treatment are listed, with at least two-fold change and *P* values less than 0.05. *n* = 14 for circulating neutrophils prior to endotoxin treatment; *n* = 17 for circulating neutrophils post endotoxin treatment; *n* = 17 for airway neutrophils post endotoxin treatment

To examine the influence of bacterial infection on CD177 expression, we downloaded another dataset GSE5772 (Tang et al., 2007) profiling neutrophil transcripts from normal individuals or septic patients with gram-negative, gram-positive, or mixed bacterial infections. We found that only septic patients with mixed bacterial infections exhibited significantly increased CD177 expression in their neutrophils; whereas septic patients infected with either gram-negative or gram-positive bacteria had a moderate but not significant increase of CD177 expression (Fig. 1C). These data indicate that CD177 expression in neutrophils can be induced by bacterial infection. Induction of CD177, however, is not necessarily mediated through toll-like receptor-mediated signaling since *in vitro* treatment with endotoxin alone failed to induce CD177 expression.

### Generation of CD177 genetic knockout mouse models

**Figure 2:**
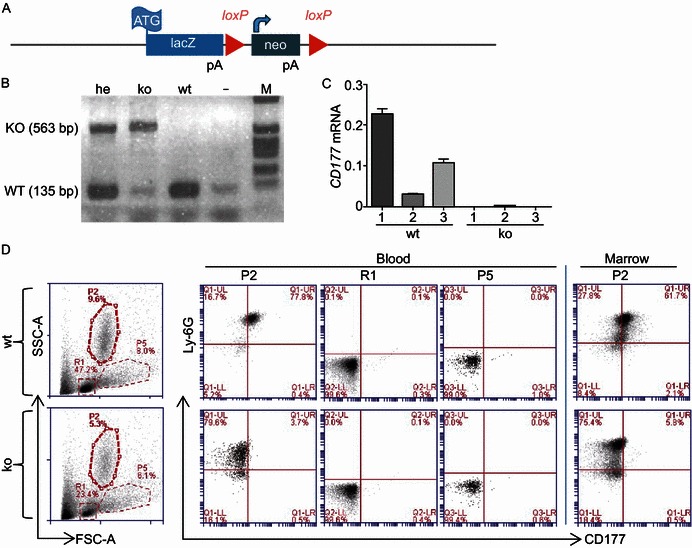
**Genetic deletion of*CD177*in mice**. (A) Schematic depiction of the *CD177* targeting construct. The entire *CD177* locus was replaced by LacZ and Neomycin by homologous recombination. (B) Image representative for genotyping CD177 genetic knock out mice. Genomic DNA was purified from tail clips and amplified with specific primers listed in the Materials and Methods. he: heterozygous knock out; ko: homozygous knock out; wt: wild type. (C) mRNA expression of *CD177* in bone marrow cells from WT or *CD177*^−/−^ mice, analyzed by real-time PCR, *n* = 3. (D) Surface expression of CD177 in blood and bone marrow cells from WT or *CD177*^−/−^ mice. Blood and bone marrow cells (marrow) were collected and stained with anti-Ly-6G-FITC, and anti-CD177-PE antibodies, followed by flow cytometry, *n* = 3

**Figure 3:**
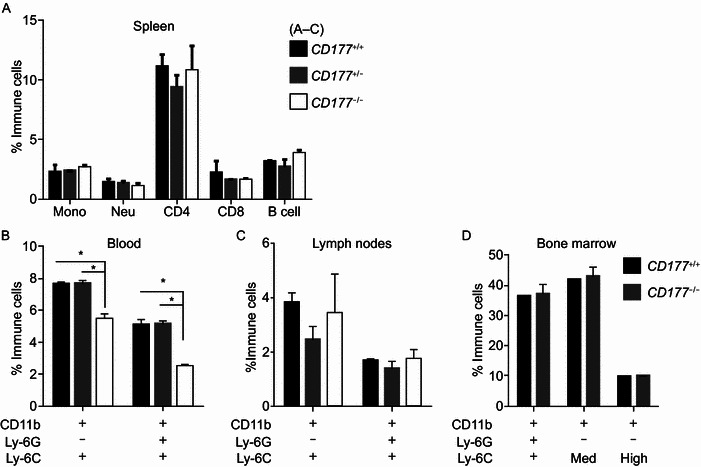
**Normal development of myeloid cell lineages in*CD177***^**−/−**^**mice**. (A) Splenic cells from seven-week-old mice were stained with anti-CD45, anti-Ly-6C, anti-Ly-6G, anti-CD11b, anti-CD11c, anti-CD3, anti-CD4, anti-CD8, and anti-B220 antibodies, followed with flow cytometry (*n* = 4–5). (B–D) Seven-week old mice were euthanized and different organs were collected. Single cell suspensions were prepared for immunolabelling with different antibodies and analyzed with flow cytometry. Blood cells (B), single cells from lymph nodes (C), or bone marrow cells (D) were labeled anti-CD45, anti-Ly-6C, anti-Ly-6G, anti-CD11b, anti-CD11c, followed with flow cytometry. CD45^+^CD11b^+^Ly-6C^+^Ly-6G^+^ cells are defined as neutrophils; CD45^+^CD11b^+^Ly-6C^+^Ly-6G^−^ cells are defined as monocytic cells. *n* = 4–5. *P < 0.05

To understand the physiological role of CD177 in neutrophils, we generated a *CD177* genetic knockout mouse by deleting the whole mouse *CD177* gene locus via homologous recombination (Fig. 2A). We developed a genotyping protocol to differentiate wild type (wt, *CD177*^*+*/*+*^), heterozygous (he, *CD177*^+/−^), or homozygous (ko, *CD177*^−/−^) animals (Fig. 2B). Ly-6G^+^ neutrophils from *CD177*^−/−^ mice lack mRNA and surface expression of CD177, indicating loss of CD177 at both the RNA and protein levels (Fig. 2C and 2D, P2 gate). As observed in human, neither lymphocytes (R1 gate) nor monocytes (P5 gate) expressed CD177 in wt mice (Fig. 2D). *CD177*^−/−^ mice were fertile and litters were born at a normal Mendelian ratio without any discernible phenotype (data not shown). We collected various tissues from the *CD177*^−/−^ mice for histological assessment and found no gross or fine histological differences between wt, *CD177*^+/−^, and *CD177*^−/−^ mice (data not shown). Immunophenotyping of different lineages of immune cells was performed by flow cytometry. There was no significant difference in T and B cell frequencies from all the compartments tested, including bone marrow, blood, thymus (data not shown), and spleen (Fig. 3A). We did not see any major defects in myeloid lineages from the different compartments (Fig. 3A–D). We did observe a significant decrease of CD11b^+^Ly-6C^+^Ly-6G^+^ circulating neutrophils from *CD177*^−/−^ mice relative to WT and *CD177*^+/−^ mice (Fig. 3B), but no difference could be detected in the spleen (Fig. 3A), lymph nodes (Fig. 3C), and bone marrow (Fig. 3D). Our results indicate that CD177 is dispensable for normal neutrophil development in mouse bone marrow or distribution within secondary lymphoid tissues, which agrees with the observation that up to 5% of individuals have normal neutrophil counts and function with no detectable CD177 expression (Matsuo et al., 2000).

### CD177-deficiency leads to decreased neutrophil accumulation early after infection

**Figure 4:**
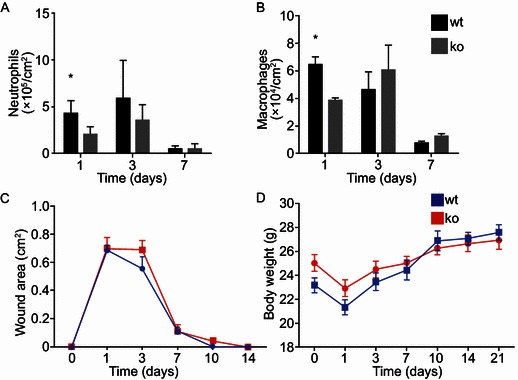
**CD177 deficiency leads to decreased neutrophil accumulation in infected skin**. (A) CD45^+^CD11b^+^Ly-6C^+^Ly-6G^+^ neutrophils in the wounded skin of *Staphylococcus Aureu-*infected mice at different days. (B) CD11b^+^F4/80^+^ macrophages in the wounded skin of *Staphylococcus Aureus*-infected mice. Wounded area (C) and body weight (D) after *Staphylococcus Aureus* infection. *P values < 0.05

To examine the role of CD177 in a bacterial infection model, we used a mouse model of *Staphylococcus aureus* skin infection. WT and *CD177*^−/−^ mice were infected with bacteria at day 0. Skin was collected from infected mice, and CD11b^+^Ly-6C^+^Ly-6G^+^ neutrophils were quantitated by flow cytometry at different days after infection. We found an initial significant decrease of neutrophils in wounded skin of *CD177*^−/−^ mice at day one after administration of *Staphylococcus aureus* (Fig. 4A). The trend was maintained at three days after infection, but the difference was not statistically significant between infected WT or *CD177*^−/−^ mice (Fig. 4A). After seven days of infection when the wound was already recovered, there was no difference in neutrophil counts between the two groups (Fig. 4A). We also observed a significant decrease of CD11b^+^Ly-6C^+^Ly-6G^-^ monocytes in the wounded skin of *CD177*^-/-^ mice at day one after infection (Fig. 4B) and there was no difference after 3 or 7 days of infection (Fig. 4B), which may reflect the importance of neutrophils in initial recruitment of monocytes. In the same model, we did not identify any significant difference in wound healing (Fig. 4C) and body weight recovery (Fig. 4D) after infection.

**Figure 5:**
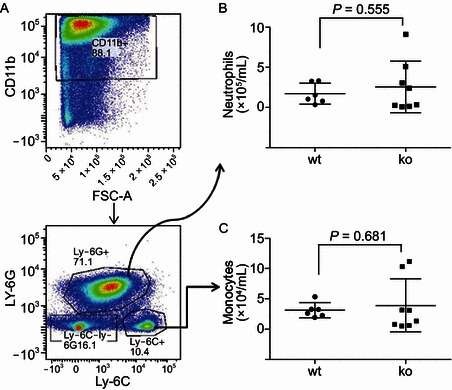
**CD177 deficiency has no impact on peritoneal accumulation of neutrophils in thioglycolate-induced peritonitis**. (A) Schematic showing flow cytometry to determine neutrophil numbers. Peritoneal cells were stained with antibodies, followed by flow cytometry to determine the total numbers of neutrophils and monocytes. (B) CD45^+^CD11b^+^Ly-6C^+^Ly-6G^+^ neutrophils were counted and graphed. (C) CD45^+^CD11b^+^Ly-6C^+^Ly-6G^-^ monocytes were graphed. *n* = 6 in WT group and *n* = 8 in *CD177*^−/−^ group. *P* values were indicated

Since it was reported that CD177^−^ and CD177^+^ neutrophils accumulate similarly in the peritoneal cavity of human peritonitis patients (Wang et al., 2013), we included a thioglycollate-induced peritonitis model to examine the effect of CD177 on neutrophil accumulation into the peritoneal cavity. Congruently, we did not find a significant difference in total peritoneal CD11b^+^Ly6C^+^Ly6G^+^ neutrophils or CD11b^+^Ly6C^+^Ly6G^−^ monocytes between WT and *CD177*^−/−^ mice (Fig. 5A and 5B), confirming that CD177 has no role in peritoneal accumulation of neutrophils in peritonitis.

### CD177-deficiency leads to increased neutrophil cell death

**Figure 6:**
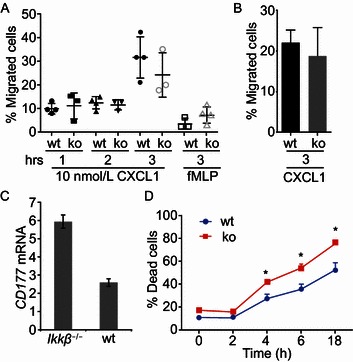
**CD177 deletion leads to neutrophil cell death**. (A and B) CD177 did not impact chemotaxis *ex vivo*. Bone marrow cells (A) or thioglycolate-induced peritoneal cells (B) were isolated from WT or *CD177*^−/−^ mice. These cells were measured for their transmigration in response to 10 nmol/L of CXCL1/KC or 100 nmol/L of fMLP at the indicated time points. Ly-6G^+^Ly-6C^+^ neutrophils counts were determined by flow cytometry. *n* = 3–4 mice per group. (C) GEO dataset GSE25211 was downloaded and analyzed for CD177 mRNA expression from purified neutrophils with (WT) or without IKKβ-deletion (*Ikkβ*^−/−^). *n* = 2 per group. (D) Primary bone marrow cells from WT or *CD177*^−/−^ mice were collected and cultured in medium. At the indicated time points, cells were stained with propidium iodide, and dead cells were enumerated by flow cytometry at each time point. *n* = 4 mice per group. *P < 0.05

Since CD177 is reported to be involved in neutrophil chemotaxis and transmigration, we examined the impact of CD177 on neutrophil chemotaxis and found that CD177-deficiency have no significant impact on CXCL1- and fMLP-induced chemotaxis when using naïve bone marrow cells (Fig. 6A), or using activated neutrophils present in thioglycolate-induced peritoneal exudate cells (Fig. 6B).

To identify the potential reason why CD177-deficiency resulted in significantly fewer neutrophils in the skin early after bacterial infection, we searched GEO profiles and found that deletion of IKKβ led to a significant increase in CD177 expression from neutrophils (Fig. 6C). IKKβ is a well-known factor whose inactivation leads to neutrophilia in the mouse and human due to increased neutrophil survival (Hsu et al., 2011). Curiously, we found that CD177-deficiency led to increased cell death of bone marrow neutrophils when cultured *ex vivo* (Fig. 6D), suggesting that CD177 could be the downstream effector of IKKβ deletion leading to increased neutrophil survival.

## DISCUSSION

Here we established a novel CD177 genetic deletion model to study its role in neutrophil biology. We found that CD177 is only expressed on neutrophils but not monocytes or lymphocytes (Fig. 2), similar to the expression patterns in human. Although CD177 had been suggested to serve as an adhesive molecule mediating migration towards chemokine gradients in human neutrophils (Sachs et al., 2007; Bayat et al., 2010; Jerke et al., 2011), we did not find any role for CD177 in promoting mouse neutrophil migration in response to CXCL1 and fMLP. Instead, we found decreased neutrophil counts in blood and infected skin from the CD177 ko animals. The CD177-deficiency led to decreased survival of bone marrow neutrophils *ex vivo*, which could potentially be used to explain the decreased neutrophil phenotype in CD177 ko mice. Among the many factors that have been shown to be involved in neutrophil death, IKKβ has attracted attention given its role in inflammatory diseases and cancer (Hsu et al., 2011). However, one of the common complications for IKKβ inhibition in patients is neutrophilia (Hsu et al., 2011), which is also observed in mice when IKKβ is deleted in the myeloid lineage. The IKKβ deletion was found to increase neutrophil life span by inducing pro-survival molecules (Hsu et al., 2011). CD177 is upregulated in neutrophils with IKKβ deletion and at the same time is involved in neutrophil survival (Fig. 6), suggesting that CD177 could be one of the factors mediating IKKβ-inhibition-induced survival and neutrophilia.

Another interesting observation is that CD177-deletion only impacts neutrophil numbers in blood and bacteria-infected skin, but not in bone marrow or secondary lymphoid tissues. These results suggest that the role of CD177 in neutrophil survival depends on the context where neutrophils are located and if other survival signals exist. It is known that neutrophils are adhesive and interact with many cell types (Geering et al., 2013). Some of the cell-cell interactions provide strong survival signal for neutrophils (Geering et al., 2013). Upon infection, however, neutrophils are recruited from the bone marrow microenvironment and lose these pro-survival signals. Importantly, certain infections lead to an upregulation of CD177 on human neutrophils (Fig. 1), leading to potentially prolonged neutrophil survival for efficient clearance of the bacteria. It is worth noting that CD177 may facilitate but is not required for survival, since there are still significant numbers of neutrophils in the blood of CD177 ko mice. Human CD177-negative neutrophils may also utilize alternative survival signals since 3%–5% of individuals who lack CD177 expression on their neutrophils have normal neutrophil counts and a normal defense towards bacteria (Stroncek et al., 2004). An interesting future avenue would be if the deletion of CD177 in human CD177-positive neutrophils has an impact on their survival under physiological or pathological conditions.

The skin infection model clearly shows that CD177 ko mice have an early decrease in neutrophils in infected skin, but the initial neutrophil defect has no significant impact on bacterial clearance as reflected by the normal wound size and body weight recovery (Fig. 5). It has been acknowledged that neutrophils, and as well as other leukocytes, need to maintain a critical threshold concentration for efficient clearance of infection (Li et al., 2002, 2004). This could explain the normal recovery in CD177 ko mice where reduced neutrophil numbers are still in sufficient quantity to eliminate *Staphylococcus aureus*.

A role for CD177 in neutrophil survival could be dependent upon particular physiological or pathological conditions. Ramirez-Velazquez et al. showed that CD177^+^ neutrophils co-expressing IL-17 are increased in moderate to severe allergic asthma (Ramirez-Velazquez et al., 2013). Our analysis based on published datasets demonstrated that CD177 expression is increased in circulating and BAL neutrophils when human volunteers were instilled with endotoxin (Fig. 1), a condition mimicking bacterial infection. All together, we believe that in certain respiratory diseases, especially those that can be exacerbated by secondary bacterial infection and involve neutrophilic inflammation (Simpson et al., 2007; Simpson et al., 2008; Essilfie et al., 2011), CD177 could be a potential contribution to the accumulation of neutrophils in the lung airway. This process, if uncontrolled, could lead to tissue damage and more severe disease. Thus CD177 may provide a target to treat respiratory diseases that are worsened by uncontrolled neutrophilic inflammation.

## MATERIALS AND METHODS

### CD177 knockout mice construction

All animals were maintained under specific pathogen-free conditions according to the IACUC guidelines. The *CD177* knockout mouse strain used for this research project was created from ES cell clone (12120a-B11), obtained from the KOMP Repository (www.komp.org) and generated by Regeneron Pharmaceuticals, Inc (Valenzuela et al., 2003). We purchased mouse sperm carrying the CD177 deletion allele from the UC Davis KOMP Repository and sent it to Jackson Laboratories for *in vitro* fertilization. Seven mice were obtained with 3 carrying a heterozygous deletion of CD177. Mice were backcrossed to C57BL/6 for 6 generations. Genotyping of the CD177 knockout mice was performed through standard PCR procedures. Primers used for genotyping are: WT allele (135 bp): forward primer 5′-GGTGATCTGGCTCAGGACAG-3′ and reverse primer 5′-CACCTGTGGGTGTAGGTAGC-3′; mutant allele (563 bp): forward primer 5′-ATTCGGCTATGACTGGGCAC-3′ and reverse primer 5′-TGAATCCAGAAAAGCGGCCA-3′.

### Flow cytometric analysis

Single-cell suspensions were prepared from spleen, bone marrow, thymus and blood, and red blood cells were lysed with red blood lysis buffer containing 155 mmol/L NH_4_Cl, 12 mmol/L NaHCO_3_ and 0.1 mmol/L EDTA. Cells were stained with different isotype controls or antibodies with different fluorophores, supplemented with CD16/CD32 FcR blockers. After being stained for 30 min, cells were washed twice and fixed in PBS containing 1% paraformaldehyde. Labeled cells were collected on a FACS Canto II (BD Biosciences) or LSR II (BD Biosciences) using Diva acquisition software, and analyzed using FlowJo (TreeStar, Stanford, CA, USA). The following antibodies were used: anti-CD3 (145-2C11, Ebiosciences), anti-CD4 (GK1.5, Ebiosciences), anti-CD8 (53-6.7, Ebiosciences), anti-B220 (RA3-6B2, Ebiosciences), CD45 (30-F11, Ebiosciences), Ly-6C (HK1.4, Ebiosciences), Ly-6G (1A8, BD Pharmingen), CD11b (M1/70, Ebiosciences), and the rat anti-mouse CD177 mAb was developed in our own laboratory.

### Thioglycollate-induced peritonitis

Eight-week old WT or *CD177*^−/−^ mice were injected i.p. with 1 mL of 4% sterile thioglycollate. Four hours later, mice were anesthetized and peritoneal cells were recovered using 10 mL of ice cold PBS and stained with an antibody cocktail including anti-CD45, anti-CD11b, anti-Ly-6C, anti-Ly-6G antibodies to quantitate peritoneal neutrophils.

### Transmigration assay

Bone marrow cells or peritoneal cells harvested after thioglycollate stimulation were seeded into the top chamber of a modified Boyden chamber with 0.5 µm pore size filter. The lower chamber included medium containing 10 nmol/L of CXCL1/KC or left untreated. Three hours later, all cells were collected and stained with anti-CD11b, anti-Ly-6C, anti-Ly-6G antibodies to quantitate neutrophil migration into the lower chamber by flow cytometry. Random neutrophil transmigration was subtracted from the CXCL1-treated chambers.

### *Staphylococcus aureus* skin infection model

A USA 100, methicillin sensitive, spa type 002 *Staphylococcus aureus* clinical isolate (347) was grown in TSB medium (1.7% enzymatic digest of casein, 0.3% enzymatic digest of soybean meal, 0.5% NaCl, 0.25% K_2_HPO_4_, 0.25% dextrose, pH 7.3) overnight at 37°C at 200 rpm. Mid-logarithmic phase bacteria were obtained after 2.5 h subculture of a 1:100 dilution of the overnight culture. Bacterial cells were pelleted and resuspended in PBS. Spectrophotometric readings of absorbance at 600 nm were used to estimate bacterial concentration, and 20 μL of *Staphylococcus aureus* at 5 × 10^7^ colony forming units was used for inoculations. Mice were anesthetized with isoflurane, abdominal skin was shaved with Accuedge microtome blades (CardinalHealth, Dublin, OH) and exposed skin was gently stroked 15 times with 200 grit sandpaper. 20 μL of bacterial suspension or PBS was applied to this surface and a gentle stream of air was aimed at the inoculation site until the inoculum suspension on the skin was dry. Finally, infected skin sites were covered with a Band Aid for 1 h.

The lesions and weight were recorded at day 0, 1, 3, 7, 10, 14 and 21 after infection. Digital photos of skin lesions were recorded and analyzed for lesion area using the Image J Software. Skin lesions were then carefully excised. Single-cell suspensions from infected skin were prepared by incubating with 0.6% trypsin for 90 min at 37°C. Trypsin-digested skin was then chopped into small pieces and incubated with 500 μg/mL collagenase type II (Gibco BRL, Grand Island, NY, USA) for 90 min at 37°C and dissociated by passing through 16, 18 and 20 gauge needles. The resulting cell suspensions were incubated for an additional 90 min at 37°C to allow for recovery of surface antigens. All samples were passed through a 70 μm filter and single cells were stained with a cocktail of antibodies including anti-CD45, anti-CD11b, anti-Ly-6C, anti-Ly-6G, anti-F4/80 antibodies and analyzed by flow cytometry. The total cell recovery of various populations was divided by the area of lesion skin and the resulting quotient was represented as cells/cm^2^.

### Microarray analysis

GEO datasets GSE2322 (Coldren et al., 2006) and GSE5772 (Tang et al., 2007) were downloaded. Expression levels of CD177 were analyzed and compared in different patient groups. GSE2322 contains 58 samples representing transcript profiles from neutrophils under different *in vitro* and *in vivo* treatments. GSE5772 has 94 samples of neutrophil transcripts from patients without sepsis, or patients with infections of gram-negative, gram-positive or mixed bacterial infections.

### Statistics

Statistical significance was determined using non-parametric Two-tailed Mann-Whitney test without assuming Gaussian distribution.

## Electronic supplementary material

Below is the link to the electronic supplementary material.

Supplementary material 1 (XLSX 268 kb)

## References

[CR1] Bayat B, Werth S, Sachs UJ, Newman DK, Newman PJ, Santoso S (2010). Neutrophil transmigration mediated by the neutrophil-specific antigen CD177 is influenced by the endothelial S536 N dimorphism of platelet endothelial cell adhesion molecule-1. J Immunol.

[CR2] Bettinotti MP, Olsen A, Stroncek D (2002). The use of bioinformatics to identify the genomic structure of the gene that encodes neutrophil antigen NB1, CD177. Clin Immunol.

[CR3] Bux J, Becker F, Seeger W, Kilpatrick D, Chapman J, Waters A (1996). Transfusion-related acute lung injury due to HLA-A2-specific antibodies in recipient and NB1-specific antibodies in donor blood. Br J Haematol.

[CR4] Caruccio L, Bettinotti M, Matsuo K, Sharon V, Stroncek D (2003). Expression of human neutrophil antigen-2a (NB1) is increased in pregnancy. Transfusion.

[CR5] Caruccio L, Bettinotti M, Director-Myska AE, Arthur DC, Stroncek D (2006). The gene overexpressed in polycythemia rubra vera, PRV-1, and the gene encoding a neutrophil alloantigen, NB1, are alleles of a single gene, CD177, in chromosome band 19q13.31. Transfusion.

[CR6] Coldren CD, Nick JA, Poch KR, Woolum MD, Fouty BW, O’Brien JM, Gruber MP, Zamora MR, Svetkauskaite D, Richter DA (2006). Functional and genomic changes induced by alveolar transmigration in human neutrophils. Am J Physiol Lung Cell Mol Physiol.

[CR7] Dillon M, Minear J, Johnson J, Lannutti BJ (2008). Expression of the GPI-anchored receptor Prv-1 enhances thrombopoietin and IL-3-induced proliferation in hematopoietic cell lines. Leuk Res.

[CR8] Essilfie AT, Simpson JL, Horvat JC, Preston JA, Dunkley ML, Foster PS, Gibson PG, Hansbro PM (2011). Haemophilus influenzae infection drives IL-17-mediated neutrophilic allergic airways disease. PLoS Pathog.

[CR9] Geering B, Stoeckle C, Conus S, Simon HU (2013). Living and dying for inflammation: neutrophils, eosinophils, basophils. Trends Immunol.

[CR10] Gohring K, Wolff J, Doppl W, Schmidt KL, Fenchel K, Pralle H, Sibelius U, Bux J (2004). Neutrophil CD177 (NB1 gp, HNA-2a) expression is increased in severe bacterial infections and polycythaemia vera. Br J Haematol.

[CR11] Goldschmeding R, van Dalen CM, Faber N, Calafat J, Huizinga TW, van der Schoot CE, Clement LT, von dem Borne AE (1992). Further characterization of the NB 1 antigen as a variably expressed 56-62 kD GPI-linked glycoprotein of plasma membranes and specific granules of neutrophils. Br J Haematol.

[CR12] Hsu LC, Enzler T, Seita J, Timmer AM, Lee CY, Lai TY, Yu GY, Lai LC, Temkin V, Sinzig U (2011). IL-1beta-driven neutrophilia preserves antibacterial defense in the absence of the kinase IKKbeta. Nat Immunol.

[CR13] Jerke U, Rolle S, Dittmar G, Bayat B, Santoso S, Sporbert A, Luft F, Kettritz R (2011). Complement receptor Mac-1 is an adaptor for NB1 (CD177)-mediated PR3-ANCA neutrophil activation. J Biol Chem.

[CR14] Kissel K, Santoso S, Hofmann C, Stroncek D, Bux J (2001). Molecular basis of the neutrophil glycoprotein NB1 (CD177) involved in the pathogenesis of immune neutropenias and transfusion reactions. Eur J Immunol.

[CR15] Kuckleburg CJ, Newman PJ (2013). Neutrophil proteinase 3 acts on protease-activated receptor-2 to enhance vascular endothelial cell barrier function. Arterioscler Thromb Vasc Biol.

[CR16] Kuckleburg CJ, Tilkens SB, Santoso S, Newman PJ (2012). Proteinase 3 contributes to transendothelial migration of NB1-positive neutrophils. J Immunol.

[CR17] Lalezari P, Murphy GB, Allen FH (1971). NB1, a new neutrophil-specific antigen involved in the pathogenesis of neonatal neutropenia. J Clin Invest.

[CR18] Li Y, Karlin A, Loike JD, Silverstein SC (2002). A critical concentration of neutrophils is required for effective bacterial killing in suspension. Proc Natl Acad Sci U S A.

[CR19] Li Y, Karlin A, Loike JD, Silverstein SC (2004). Determination of the critical concentration of neutrophils required to block bacterial growth in tissues. J Exp Med.

[CR20] Martini M, Teofili L, Larocca LM (2006). Overexpression of PRV-1 gene in polycythemia rubra vera and essential thrombocythemia. Methods Mol Med.

[CR21] Matsuo K, Lin A, Procter JL, Clement L, Stroncek D (2000). Variations in the expression of granulocyte antigen NB1. Transfusion.

[CR22] Michiels JJ, Bernema Z, Van Bockstaele D, De Raeve H, Schroyens W (2007). Current diagnostic criteria for the chronic myeloproliferative disorders (MPD) essential thrombocythemia (ET), polycythemia vera (PV) and chronic idiopathic myelofibrosis (CIMF). Pathol Biol (Paris).

[CR23] Ramirez-Velazquez C, Castillo EC, Guido-Bayardo L, Ortiz-Navarrete V (2013). IL-17-producing peripheral blood CD177 + neutrophils increase in allergic asthmatic subjects. Allergy Asthma Clin Immunol.

[CR24] Sachs UJ, Andrei-Selmer CL, Maniar A, Weiss T, Paddock C, Orlova VV, Choi EY, Newman PJ, Preissner KT, Chavakis T (2007). The neutrophil-specific antigen CD177 is a counter-receptor for platelet endothelial cell adhesion molecule-1 (CD31). J Biol Chem.

[CR25] Simpson JL, Grissell TV, Douwes J, Scott RJ, Boyle MJ, Gibson PG (2007). Innate immune activation in neutrophilic asthma and bronchiectasis. Thorax.

[CR26] Simpson JL, Powell H, Boyle MJ, Scott RJ, Gibson PG (2008). Clarithromycin targets neutrophilic airway inflammation in refractory asthma. Am J Respir Crit Care Med.

[CR27] Sirhan S, Lasho TL, Elliott MA, Tefferi A (2005). Neutrophil polycythemia rubra vera-1 expression in classic and atypical myeloproliferative disorders and laboratory correlates. Haematologica.

[CR28] Stroncek DF (2007). Neutrophil-specific antigen HNA-2a, NB1 glycoprotein, and CD177. Curr Opin Hematol.

[CR29] Stroncek DF, Jaszcz W, Herr GP, Clay ME, McCullough J (1998). Expression of neutrophil antigens after 10 days of granulocyte-colony-stimulating factor. Transfusion.

[CR30] Stroncek DF, Caruccio L, Bettinotti M (2004). CD177: a member of the Ly-6 gene superfamily involved with neutrophil proliferation and polycythemia vera. J Transl Med.

[CR31] Subramanian A, Tamayo P, Mootha VK, Mukherjee S, Ebert BL, Gillette MA, Paulovich A, Pomeroy SL, Golub TR, Lander ES (2005). Gene set enrichment analysis: a knowledge-based approach for interpreting genome-wide expression profiles. Proc Natl Acad Sci U S A.

[CR32] Tang BM, McLean AS, Dawes IW, Huang SJ, Lin RC (2007). The use of gene-expression profiling to identify candidate genes in human sepsis. Am J Respir Crit Care Med.

[CR33] Valenzuela DM, Murphy AJ, Frendewey D, Gale NW, Economides AN, Auerbach W, Poueymirou WT, Adams NC, Rojas J, Yasenchak J (2003). High-throughput engineering of the mouse genome coupled with high-resolution expression analysis. Nat Biotechnol.

[CR34] Wang L, Ge S, Agustian A, Hiss M, Haller H, von Vietinghoff S (2013). Surface receptor CD177/NB1 does not confer a recruitment advantage to neutrophilic granulocytes during human peritonitis. Eur J Haematol.

